# Supplementary material for: Comparative Analysis of Tylosema esculentum Mitochondrial DNA Revealed Two Distinct Genome Structures
Source: Biology (Basel). 2023 Sep 16;12(9):1244. doi: 10.3390/biology12091244 (PMC10525999; doi:10.3390/biology12091244)
Supplement: Supplementary file 1 [file biology-12-01244-s001.zip › biology-2553184-supplementary.pdf]

Supplementary Materials

Table S1. Summary of WGS Illumina data and sources of the 84 samples

Sample	Plant source	Raw reads	Raw data	Mito Cvrg	Cytotype
M17	NM Farm Seeds	96750044	14512506600	2480	Type 2
S_35	NM Farm Seeds	86626894	12994034100	1928	Type 2
S_19	NM Farm Seeds	55375806	8306370900	1493	Type 2
S_4	NM Farm Seeds	73435564	11015334600	2817	Type 2
S_13	NM Farm Seeds	217730028	32659504200	5368	Type 2
S_27	NM Farm Seeds	107264134	16089620100	349	Type 2
S_20	NM Farm Seeds	55371622	8305743300	1455	Type 2
S_30	NM Farm Seeds	106501502	15975225300	2963	Type 2
S_33	NM Farm Seeds	130811500	19621725000	4788	Type 2
M7	NM Farm Seeds	172383630	25857544500	4279	Type 2
M8	NM Farm Seeds	123934796	18590219400	3713	Type 2
M1 #	NM Farm Seeds	170816890	25622533500	5240	Type 2
M2	NM Farm Seeds	194335620	29150343000	933	Type 2
M11	NM Farm Seeds	125005082	18750762300	4213	Type 2
M12	NM Farm Seeds	128360580	19254087000	1500	Type 2
M15	NM Farm Seeds	134439570	20165935500	3781	Type 2
M16	NM Farm Seeds	132448260	19867239000	3789	Type 2
M23	NM Farm Seeds	134475978	20171396700	3642	Type 2
M22	NM Farm Seeds	111447592	16717138800	3891	Type 2
M24	NM Farm Seeds	127392478	19108871700	3546	Type 2
M26	NM Farm Seeds	225925198	33888779700	699	Type 2
M28	NM Farm Seeds	193804308	29070646200	4799	Type 2
M25	NM Farm Seeds	145937916	21890687400	3860	Type 2
N29	NM Farm Seeds	147710832	22156624800	4863	Type 2
M31	NM Farm Seeds	177899436	26684915400	3647	Type 2
M34	NM Farm Seeds	124221640	18633246000	1060	Type 2
M36	NM Farm Seeds	125524008	18828601200	2242	Type 2
M37	NM Farm Seeds	185151336	27772700400	6847	Type 2
M38	NM Farm Seeds	168052326	25207848900	4617	Type 2
M40	Namibia Unknown	135702768	20355415200	1720	Type 1
Index1 #	UP Farm	41499124	4149912400	269	Type 2
Index10	UP Farm Seeds	36382172	3638217200	209	Type 2
Index11	UP Farm Seeds	34631932	3463193200	295	Type 2
Index12 #	UP Farm	39339004	3933900400	804	Type 2
Index19 #	UP Farm	35994474	3599447400	258	Type 2
Index3 #	UP Farm	35991990	3599199000	395	Type 2
Index5 #	UP Farm	34349602	3434960200	814	Type 2

Index8	Namibia Unknown	42747718	4274771800	359	Type 1
Index9 #	UP Farm	34312880	3431288000	793	Type 2
R1R2	Unknown	358941018	35894101800	1550	Type 2
A1 #	Aminuis Seeds	93324222	13998633300	707	Type 1
A2	Aminuis Seeds	93445492	14016823800	437	Type 1
A3	Aminuis Seeds	84081976	12612296400	855	Type 1
A4	Aminuis Seeds	60965538	9144830700	607	Type 1
A5	Aminuis Seeds	91044746	13656711900	587	Type 1
A6	Aminuis Seeds	89785050	13467757500	677	Type 1
A7	Aminuis Seeds	86000118	12900017700	528	Type 1
A8	Aminuis Seeds	57087632	8563144800	549	Type 1
A9 #	Aminuis	62849376	9427406400	559	Type 1
A10 #	Aminuis	75633554	11345033100	541	Type 1
A11 #	Aminuis	92851730	13927759500	923	Type 1
A12 #	Aminuis	84374620	12656193000	561	Type 1
A13 #	Aminuis	83450100	12517515000	864	Type 1
nar15 #	Aminuis	81618952	12242842800	389	Type 1
nar16	UP Farm Seeds	96595344	14489301600	807	Type 2
S1 #	Tsjaka	10358444	1035844400	25	Type 1
S2 #	Tsjaka	20343934	2034393400	198	Type 1
S3 #	Tsjaka	33100100	3310010000	597	Type 1
S4 #	Tsjaka	25428338	2542833800	373	Type 1
S5 #	Okamatapati	27183834	2718383400	396	Type 1
S6 #	Tsumkwe	24185808	2418580800	419	Type 1
S7 #	Tsumkwe	22075112	2207511200	162	Type 1
S8 #	Tsumkwe	16337544	1633754400	163	Type 1
S9 #	Aminuis	25274640	2527464000	270	Type 1
S10 #	Aminuis	28329944	2832994400	311	Type 1
S11 #	Aminuis	26741342	2674134200	288	Type 1
S12 #	Aminuis	29520092	2952009200	423	Type 1
S13 #	Aminuis	NA	NA	236	Type 1
S14 #	Aminuis	22928100	2292810000	283	Type 1
S15 #	Aminuis	22856344	2285634400	486	Type 1
S16 #	Aminuis	25333752	2533375200	338	Type 1
S17 #	Aminuis	23418786	2341878600	258	Type 1
S18 #	Tsumkwe	25764102	2576410200	234	Type 1
S19 #	Aminuis	24407080	2440708000	421	Type 1
S20 #	Osire	25692398	2569239800	407	Type 1
S21 #	Osire	25940358	2594035800	381	Type 1
S22 #	Osire	32107310	3210731000	609	Type 1
S23 #	Osire	35844166	3584416600	697	Type 1
S24 #	Tsumkwe	31973008	3197300800	411	Type 1
S25 #	Ombujondjou	24401422	2440142200	539	Type 1

S26 #	Ombujondjou	32758062	3275806200	433	Type 1
S27 #	Epukiro	28122544	2812254400	573	Type 1
S28 #	Epukiro	31273940	3127394000	699	Type 1
S29 #	Otjiwarongo	25274640	2527464000	498	Type 1

43 independent samples for sequence diversity and phylogenetic analysis; Mito Cvrg = approximate read depth for single-copy regions of the mitogenome

Table S2. List of homologous fragments between the mitochondrial and chloroplast genomes of *Tylosema esculentum*.

Plastome		Mitogenome			Alignment		
Start	End	Chr	Start	End	Length	%Identity	E-value
260	339	LS1	128344	128265	80	98.75	4.66E-33
24312	24387	LS1	142158	142233	76	98.684	7.80E-31
26169	26237	LS2	66608	66676	69	100	1.31E-28
31434	31539	LS1	238735	238628	108	93.519	4.63E-38
34586	34943	LS2	14640	14299	360	83.056	4.34E-83
35308	45222	LS2	136516	146313	9926	98.146	0
38063	38123	LS2	139272	139214	61	88.525	6.20E-12
45221	45257	LS2	146312	35	37	100	8.02E-11
54011	54085	LS1	59870	59796	75	94.667	2.83E-25
54818	54845	LS2	130707	130734	28	100	8.08E-06
67414	67689	LS2	84625	84353	282	90.426	1.53E-97
98539	98610	LS2	80134	80205	72	97.222	6.07E-27
102961	103824	LS2	20757	19899	891	74.074	1.21E-83
106303	106383	LS2	43447	43375	81	90.123	1.02E-19
106303	106383	LS2	75967	75895	81	90.123	1.02E-19
110921	111003	LS2	74229	74312	84	97.619	4.66E-33
136648	136730	LS2	74312	74229	84	97.619	4.66E-33
141268	141348	LS2	43375	43447	81	90.123	1.02E-19
141268	141348	LS2	75895	75967	81	90.123	1.02E-19
143827	144690	LS2	19899	20757	891	74.074	1.21E-83
149041	149112	LS2	80205	80134	72	97.222	6.07E-27

Table S3. Potential effect of variations found in the gene sequences on the 9,798 bp cpDNA insertion in the 84 individuals.

**Position**	**Reference**	**Variation**	**Gene**	**AA Substitution**
35570	A	C	*psbC*	N44H
35634	G	T	*psbC*	G65V

35774	TTTGTGTCT	DEL	*psbC*	FVS112-114Δ
35884	TTATGTAT	DEL	*psbC*	Y149fs*
36347	GGACCTACT	DEL	*psbC*	GPT303-305Δ
38753	CGATCTTC	DEL	*rps14*	G67fs*
38867	G	T	*rps14*	synonymous
38873	TTTTTTTGAGGATCGGCGAA	DEL	*rps14*	I23fs*
38893	T	G	*rps14*	I23L
38922	CTTTTCTTCT	DEL	*rps14*	E10fs*
39144	A	G	*psaB*	V711A
39264	CAATATCCA	DEL	*psaB*	GYW 669-671Δ
39412	C	A	*psaB*	D622Y
39414	C	A	*psaB*	R621I
39429	A	C	*psaB*	L616W
40061	G	T	*psaB*	D405E
40517	A	G	*psaB*	synonymous
40544	A	C	*psaB*	F244L
41030	A	G	*psaB*	synonymous
41243	A	G	*psaB*	synonymous
41716	C	A	*psaA*	D615Y
41718	G	T	*psaA*	S614Δ*
42060	GTTGCACCAGGGGCC	DEL	*psaA*	APGAT491-495Δ
42385	C	T	*psaA*	V392M
42587	C	T	*psaA*	synonymous
42793	G	C	*psaA*	Q256E
42817	CCAAAAGAT	DEL	*psaA*	DLL245-247Δ
42838	G	T	*psaA*	L241I
43508	ATCCCTAT	DEL	*psaA*	A15fs*

Genetic info represented by symbols in the table: AA, amino acids; Δ, deletion; fs, frameshift; *, nonsense mutation.

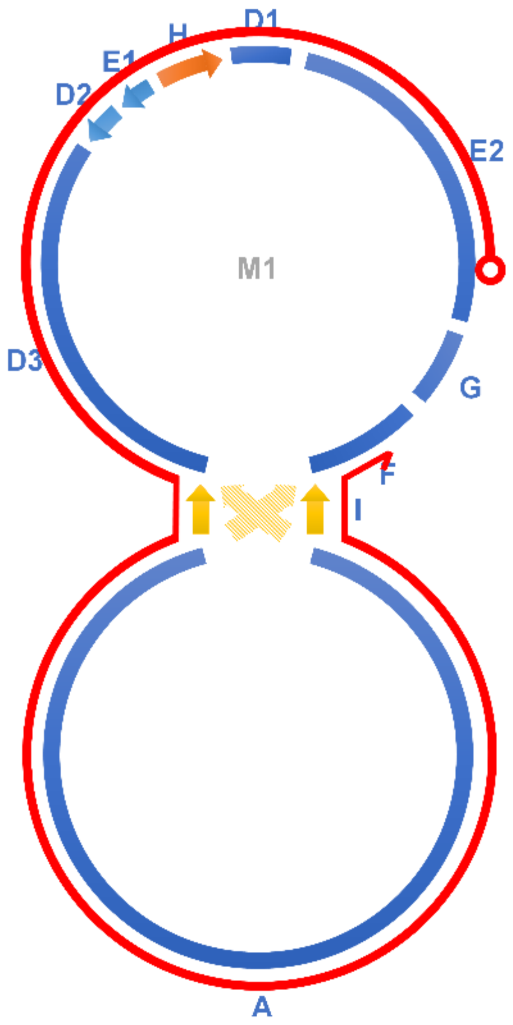

Figure S1. Alignment of contig tig00000040 of 159,605 bp, assembled by HiCanu on the Sample 4 PacBio HiFi reads, to the type 2 marama subgenomic chromosome M1. The Canu input genome size was set to 2M to better capture complete organelle genome sequences. The red curve indicates where the contig is aligned, starting from the red circle and ending with the red arrow.

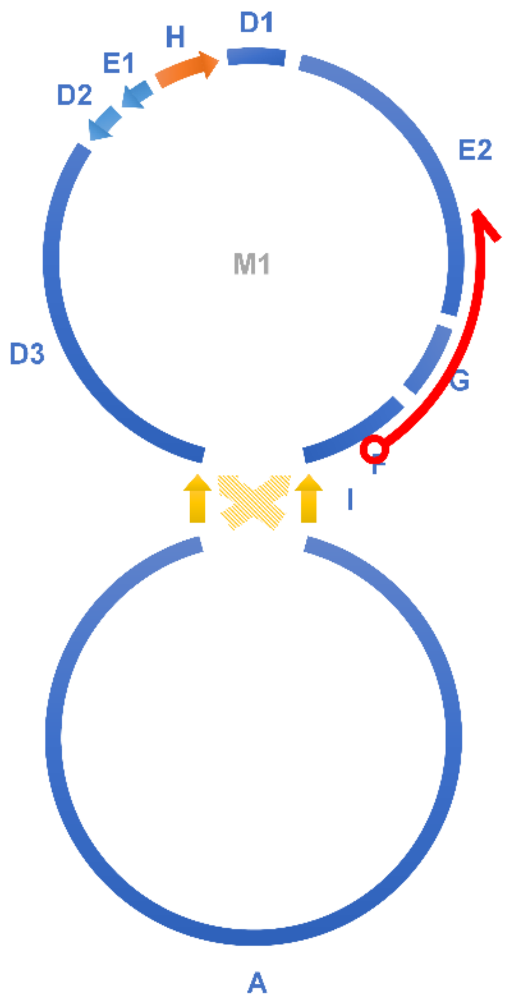

Figure S2. Alignment of contig tig00000050 of 13,828 bp, assembled by HiCanu on the Sample 4 PacBio HiFi reads, to the type 2 marama subgenomic chromosome M1. The Canu input genome size was set to 2M to better capture complete organelle genome sequences. The red curve indicates where the contig is aligned, starting from the red circle and ending with the red arrow. This together with tig00000040 (Figure S1) verifies the structure of M1. Discontinuous assembly results from fluctuating in coverage at some locations, and the complete structure has been verified by manual extension.

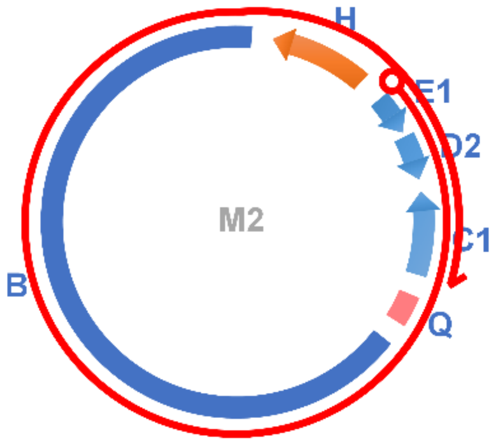

Figure S3. Alignment of contig tig00000055 of 64,325 bp, assembled by HiCanu on the Sample 4 PacBio HiFi reads, to the type 2 marama subgenomic chromosome M2. The Canu input genome size was set to 2M to better capture complete organelle genome sequences. The red curve indicates where the contig is aligned, starting from the red circle and ending with the red arrow. This contig validates the circular structure of M2.

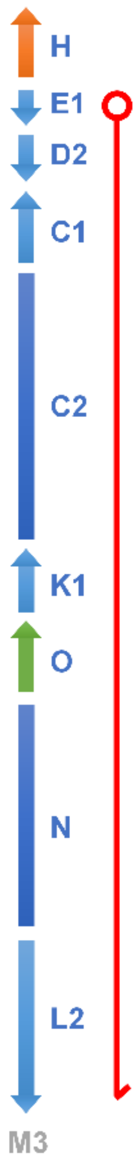

Figure S4. Alignment of contig tig00000057 of 87,648 bp, assembled by HiCanu on the Sample 4 PacBio HiFi reads, to the type 2 marama subgenomic chromosome M3. The Canu input genome size was set to 2M to better capture complete organelle genome sequences. The red line indicates where the contig is aligned, starting from the red circle and ending with the red arrow.

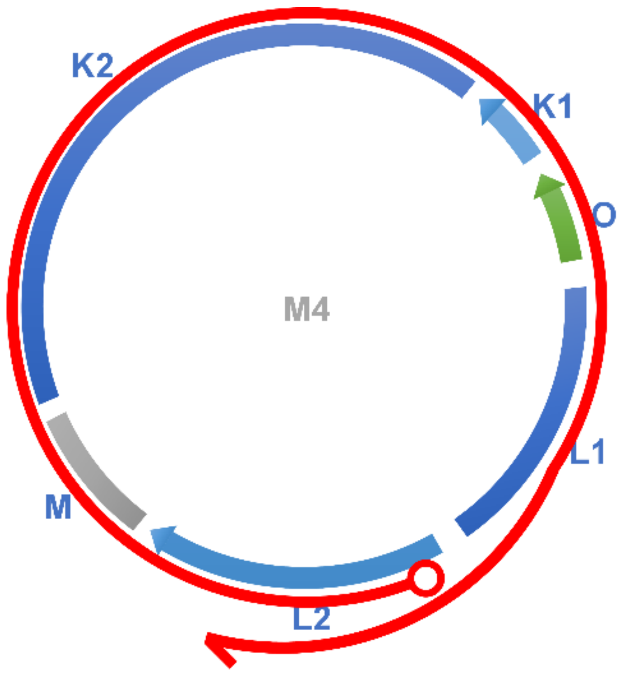

Figure S5. Alignment of contig tig00000058 of 123,793 bp, assembled by HiCanu on the Sample 4 PacBio HiFi reads, to the type 2 marama subgenomic chromosome M4. The Canu input genome size was set to 2M to better capture complete organelle genome sequences. The red curve indicates where the contig is aligned, starting from the red circle and ending with the red arrow. This verifies the circular structure of M4.

Two different connections were seen at the pair of inverted repeats I, which formed a normal circular molecule, or an 8-shaped ring resulting from recombination on the repeats (A^{1}-I-D3 and $A^{82,874}$-I-F became $A^{82,874}$-I-D3 and A^{1}-I-F). Both molecules were confirmed by the PacBio HiFi reads of Sample 4 and found to be in very close proportions, as shown in Figure S6-9.

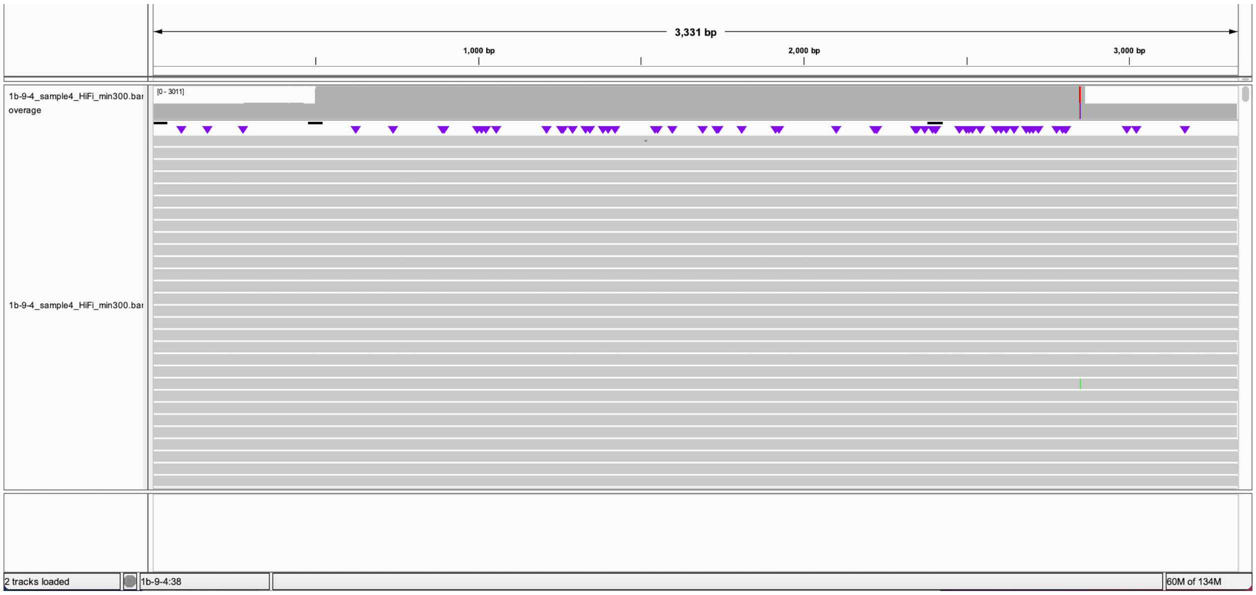

Figure S6. Alignment of Sample 4 PacBio HiFi reads to the artificial chromosome concatenated by node A (82,375 - 82,874 bp), node I, and node D3 (26,671-26,172 bp) using pbmm2. The minimum length was set to 300 to avoid interference from homologous DNA fragments. The coverage of the inverted repeat node I was doubled as expected.

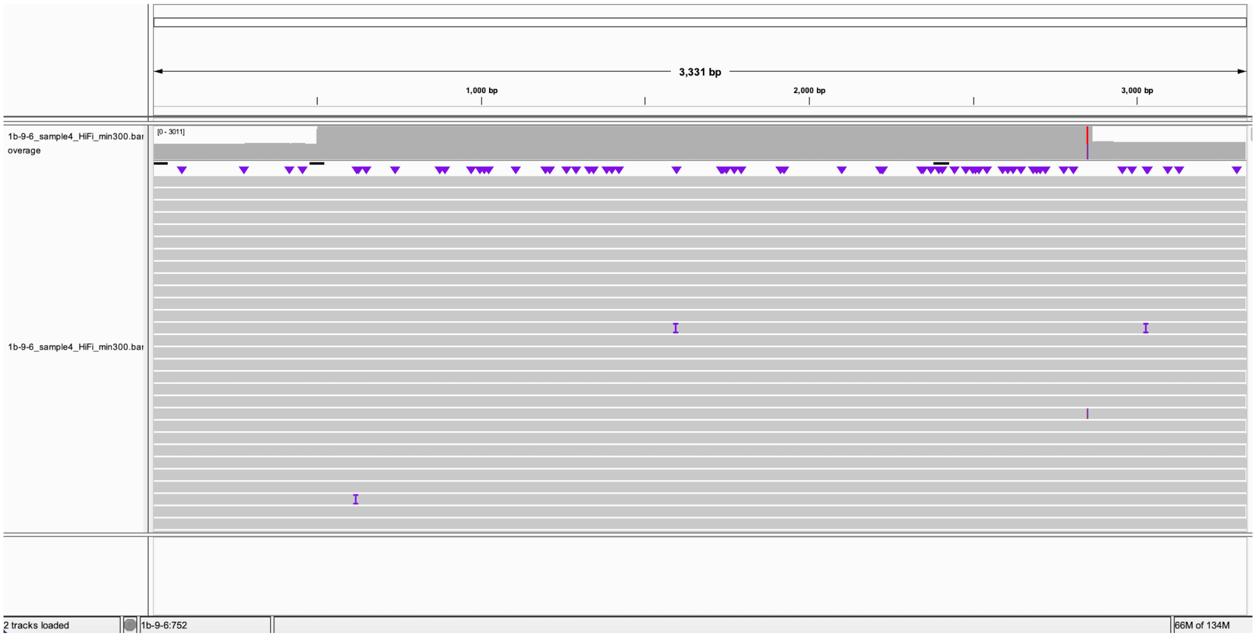

Figure S7. Alignment of Sample 4 PacBio HiFi reads to the artificial chromosome concatenated by node A (82,375 - 82,874 bp), node I, and node F (8,590 – 8,091 bp) using pbmm2. The minimum length was set to 300 to avoid interference from homologous DNA fragments. The coverage of the inverted repeat node I was doubled as expected.

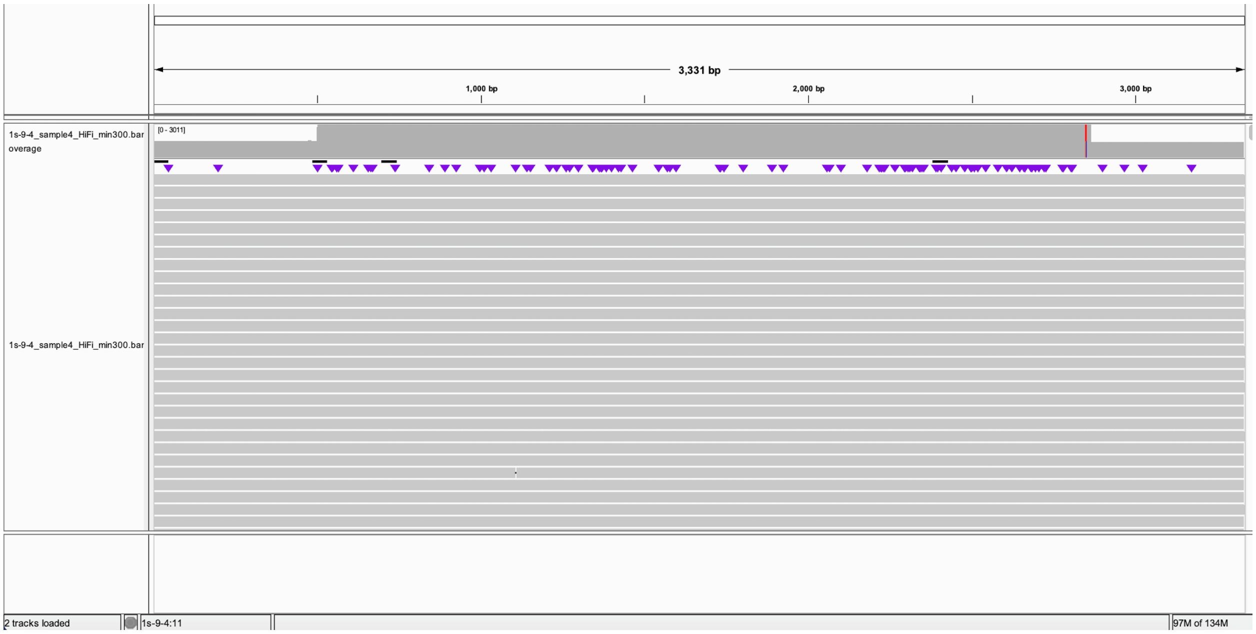

Figure S8. Alignment of Sample 4 PacBio HiFi reads to the artificial chromosome concatenated by node A (500 - 1 bp), node I, and node D3 (26,671-26,172 bp) using pbmm2. The minimum length was set to 300 to avoid interference from homologous DNA fragments. The coverage of the inverted repeat node I was doubled as expected.

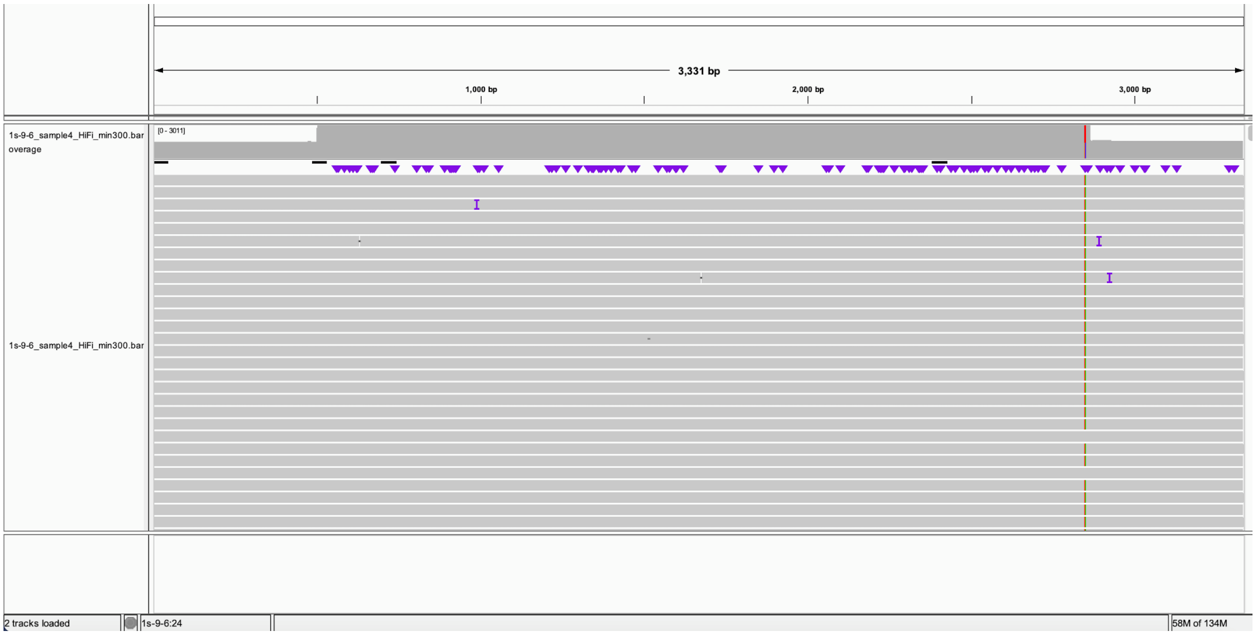

Figure S9. Alignment of Sample 4 PacBio HiFi reads to the artificial chromosome concatenated by node A (500 - 1 bp), node I, and node F (8,590 – 8,091 bp) using pbmm2. The minimum length was set to 300 to avoid interference from homologous DNA fragments. The coverage of the inverted repeat node I was doubled as expected.

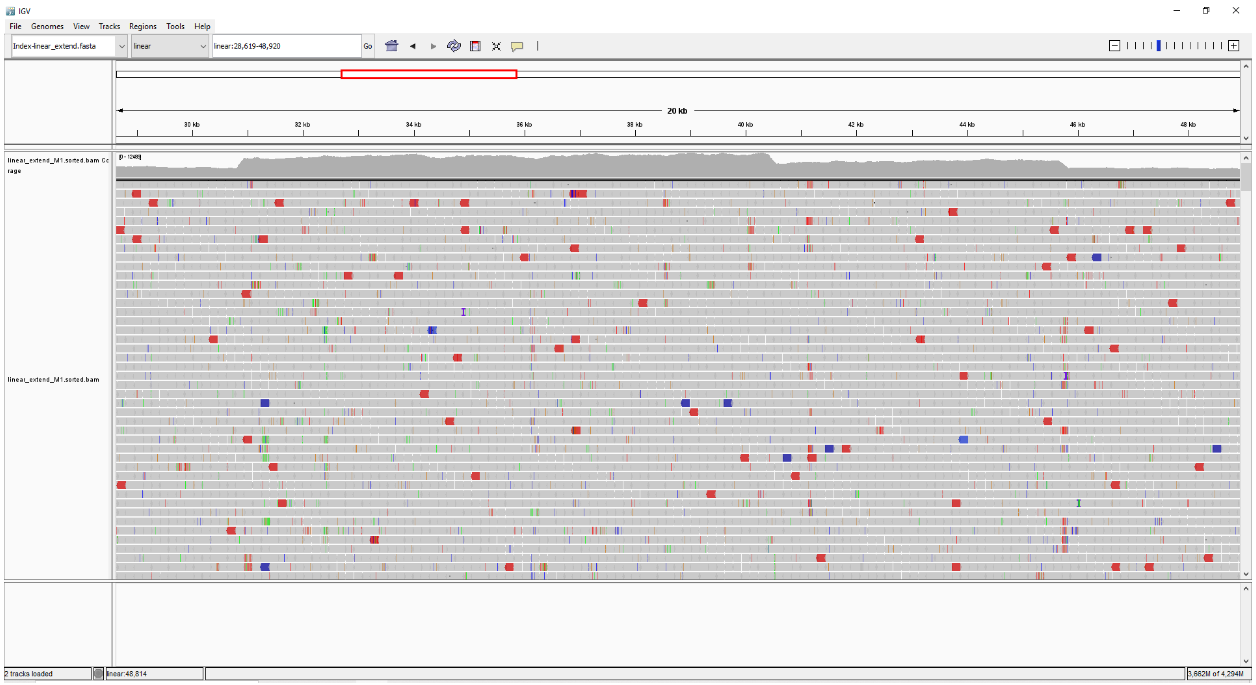

Figure S10. IGV visualization of WGS Illumina reads of individual M1 aligned to the one end of the linear chromosome M3 (extended by the sequence of D1, thus arranged as D1-H-E1-D2-C1-C2). A clear increase of coverage from one copy to two, and then to three can be seen from right to left. The two ends, C2 and D1 have only one copy. C1 has two copies, as it is owned by both chromosomes M2 and M3. H-E1-D2 is a long repeat present in three chromosomes, M1, M2, and M3, with tripled depth as shown.

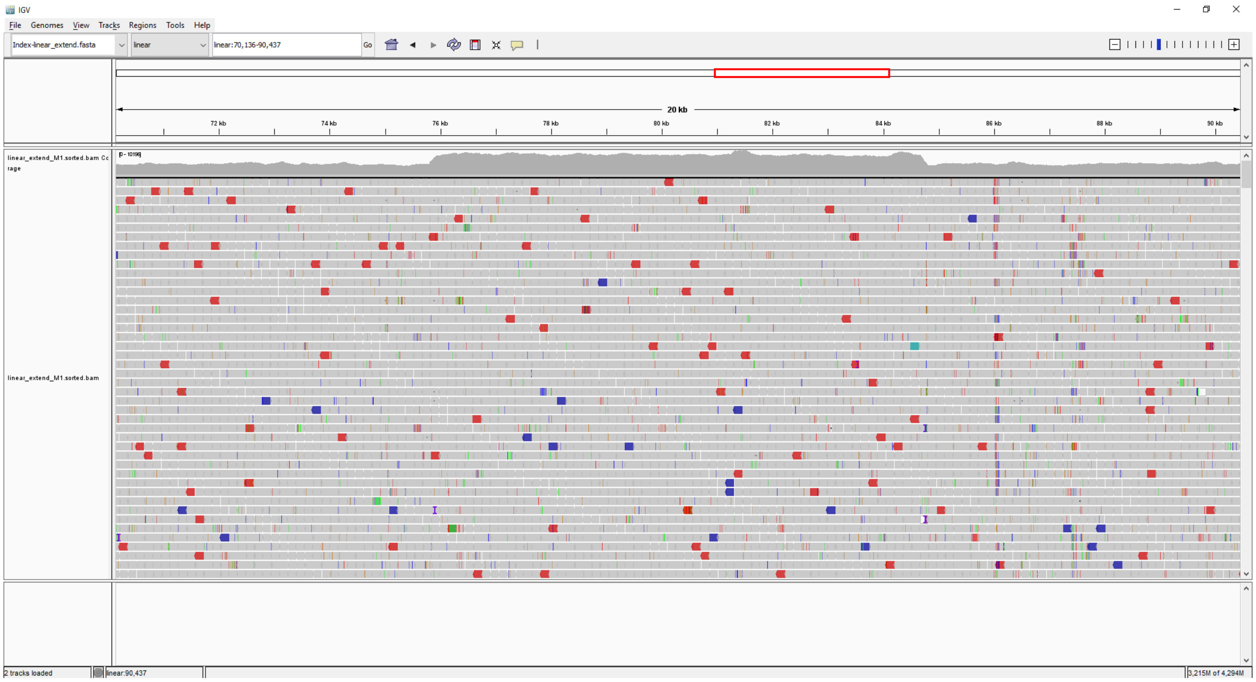

Figure S11. IGV visualization of WGS Illumina reads of individual M1 aligned to the mitochondrial genome fragment C2-K1-O-N via Bowtie2. The coverage of K1 and O in the middle is doubled because it is a long repeat owned by two chromosomes, M3 and M4.

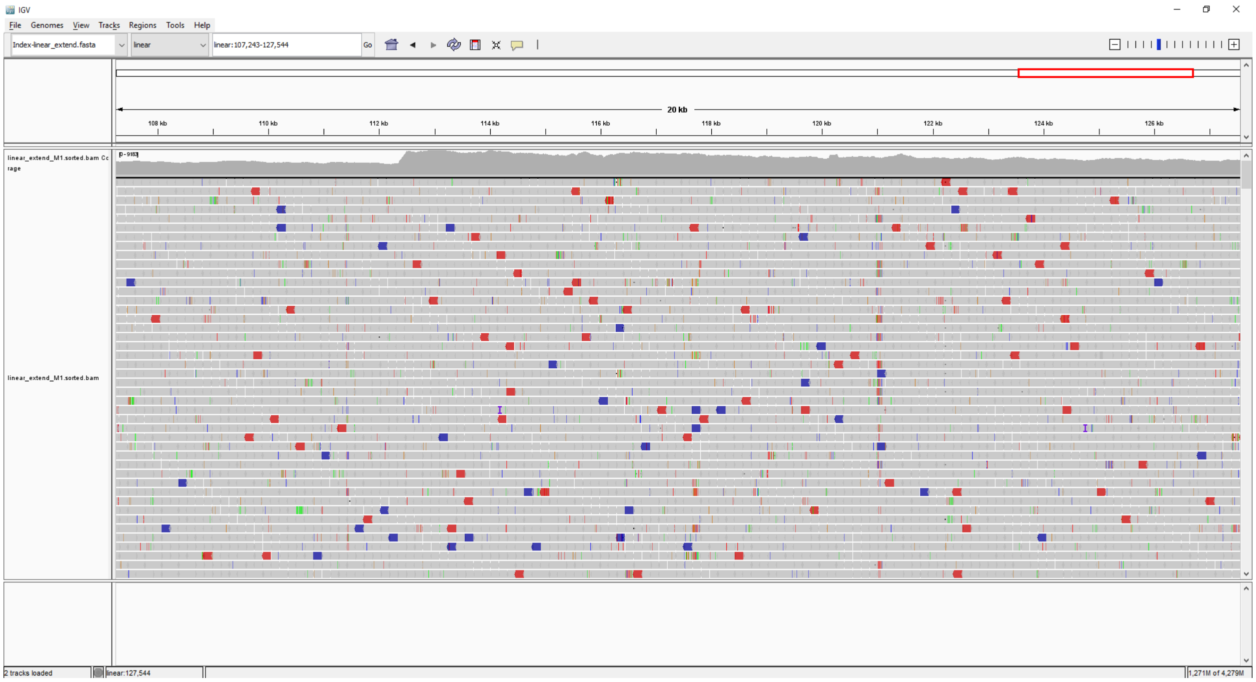

Figure S12. IGV visualization of WGS Illumina reads of marama individual M1 aligned to the mitochondrial genome fragment L1-L2 by Bowtie2. From L2 onwards, the read depth is doubled because L2 is a segment owned by both chromosomes M3 and M4. However, the coverage gradually decreases from left to right after doubling, as the end of the linear chromosome M3 is reached.

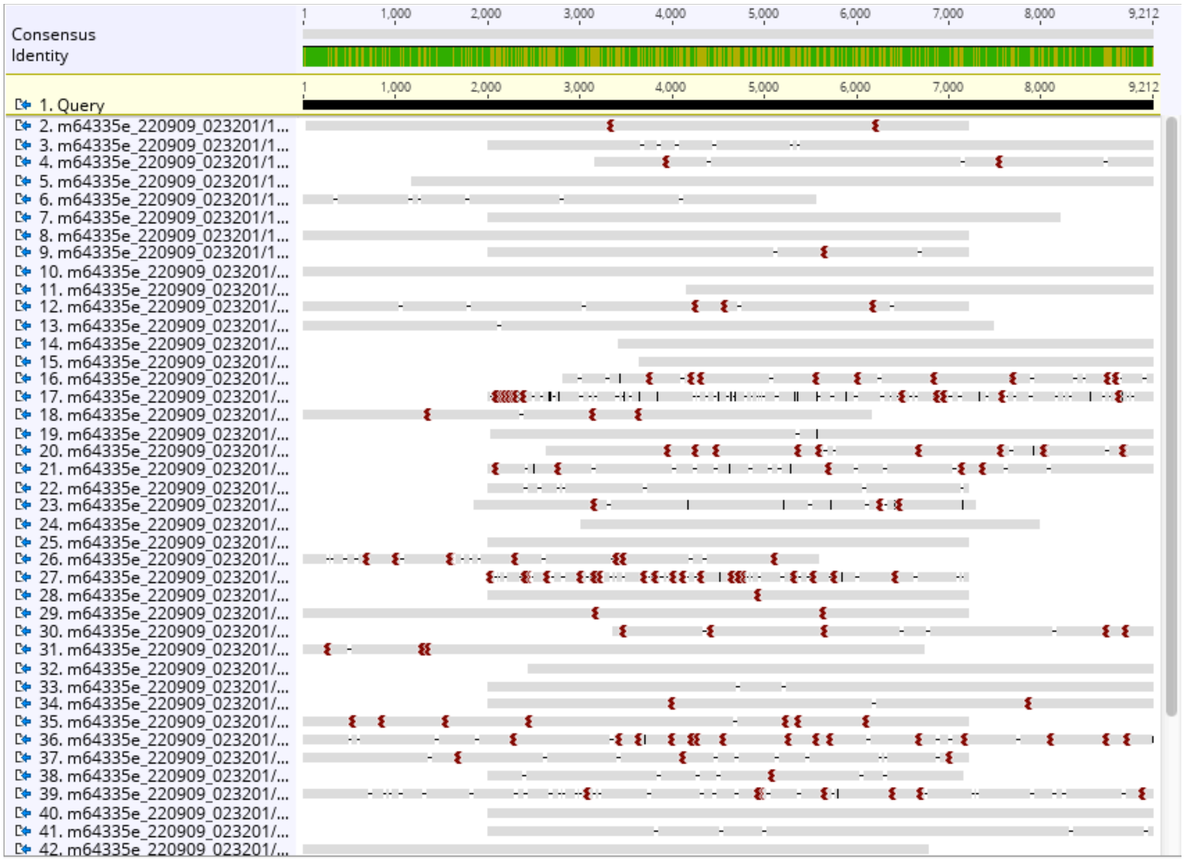

Figure S13. The PacBio HiFi reads of the type 1 individual Sample 32 were mapped to the fragment B-H-B (2000 bp sequences were taken from both ends of node B, with node H in the middle) by BLAST in Geneious 9. Reads going across the entire fragment B-H-B can be seen, indicating the presence of a closed ring consisting only of nodes B and H in type 1 individual Sample 32. In addition, reads from chromosome M1, mapped only to node H, were also observed. Reads, mapped to node H and one end of node B (the other end entered chromosome M1, not shown here), were seen, representing a combination of the two subgenomic structures.

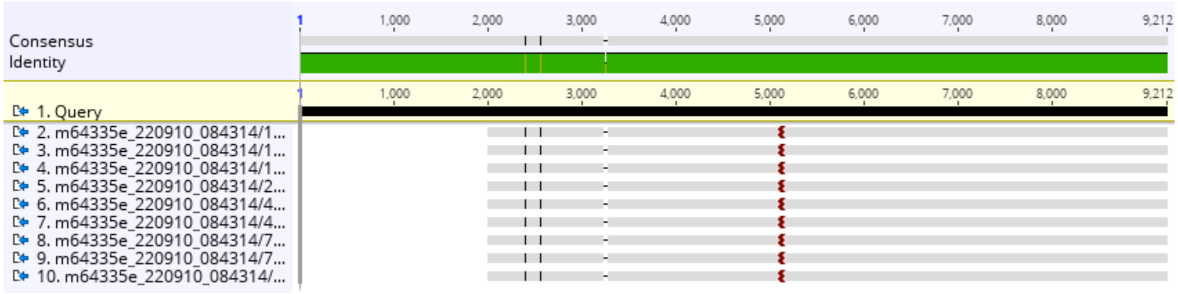

Figure S14. The PacBio HiFi reads of the type 2 individual Sample 4 were mapped to the fragment B-H-B (2000 bp sequences were taken from both ends of node B, with node H in the middle) by BLAST in Geneious 9. Node H was only connected to one end of node B but not to the other. The closed ring consisting only of node B and H does not exist in type 2 individual Sample 4.

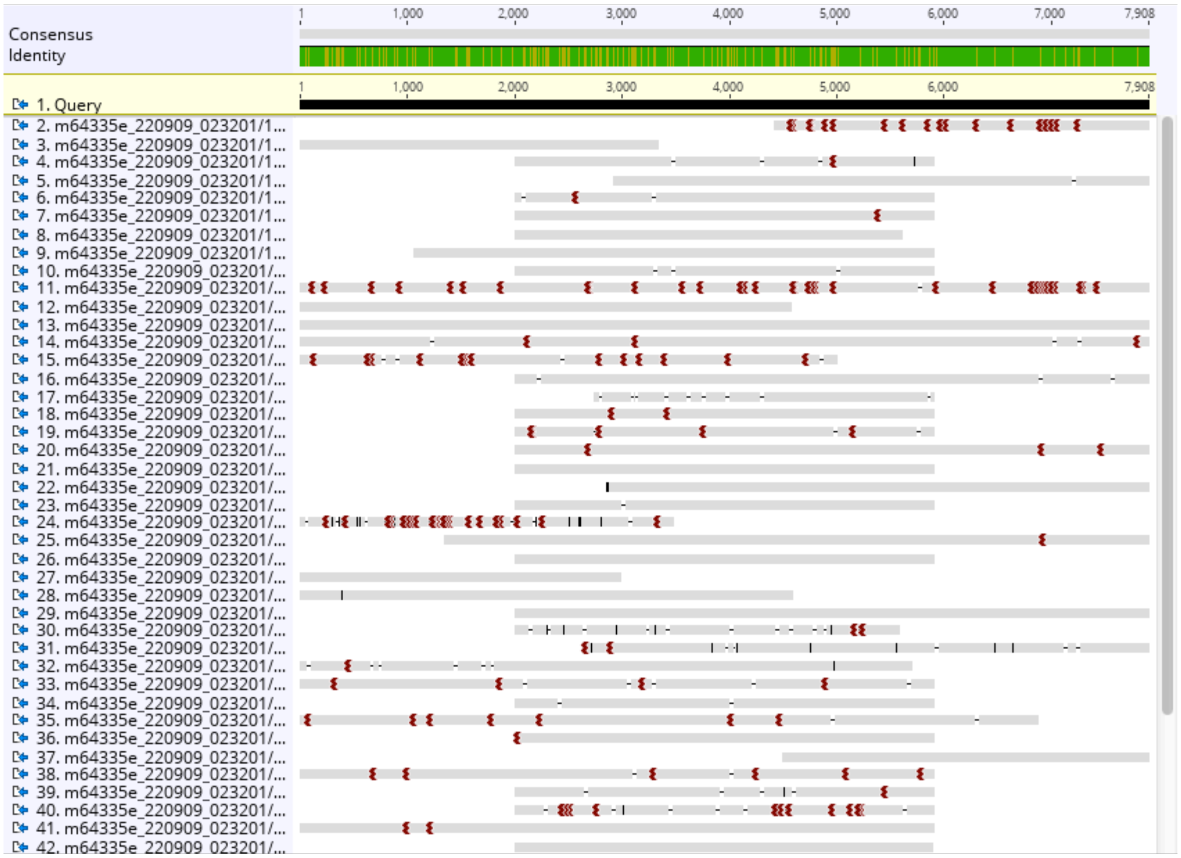

Figure S15. The PacBio HiFi reads of the type 1 individual Sample 32 were mapped to the fragment C-A-C (node A, 39,444-43,351 bp, surrounded by 2000 bp sequences at both ends of node C) by BLAST in Geneious 9. Reads spanning the entire fragment C-A-C can be seen, indicating the existence of a closed subgenomic ring consisting only of nodes C and A in type 1 individual Sample 32. Furthermore, reads from chromosome M1 were observed to map only to node A in the middle, and also reads were seen to map to node A and one end of node C (the other end entered chromosome M1, not shown here), representing a combination of the two subgenomic structures.

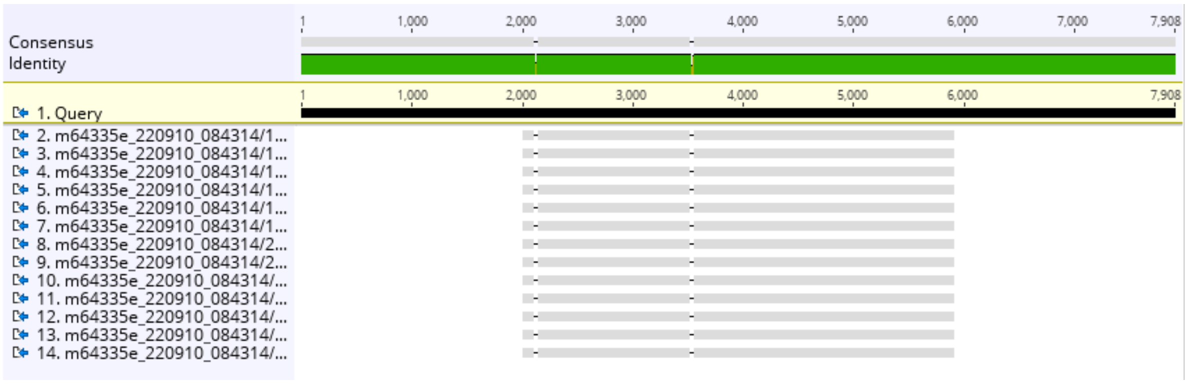

Figure S16. The PacBio HiFi reads of the type 2 individual Sample 4 were mapped to the fragment C-A-C (node A, 39,444-43,351 bp, surrounded by 2000 bp sequences at both ends of node C) by BLAST in Geneious 9. Node A (39,444-43,351 bp) was not connected to either end of node C in Sample 4, suggesting that the two types of mitochondrial genomes have distinct structures in this region.

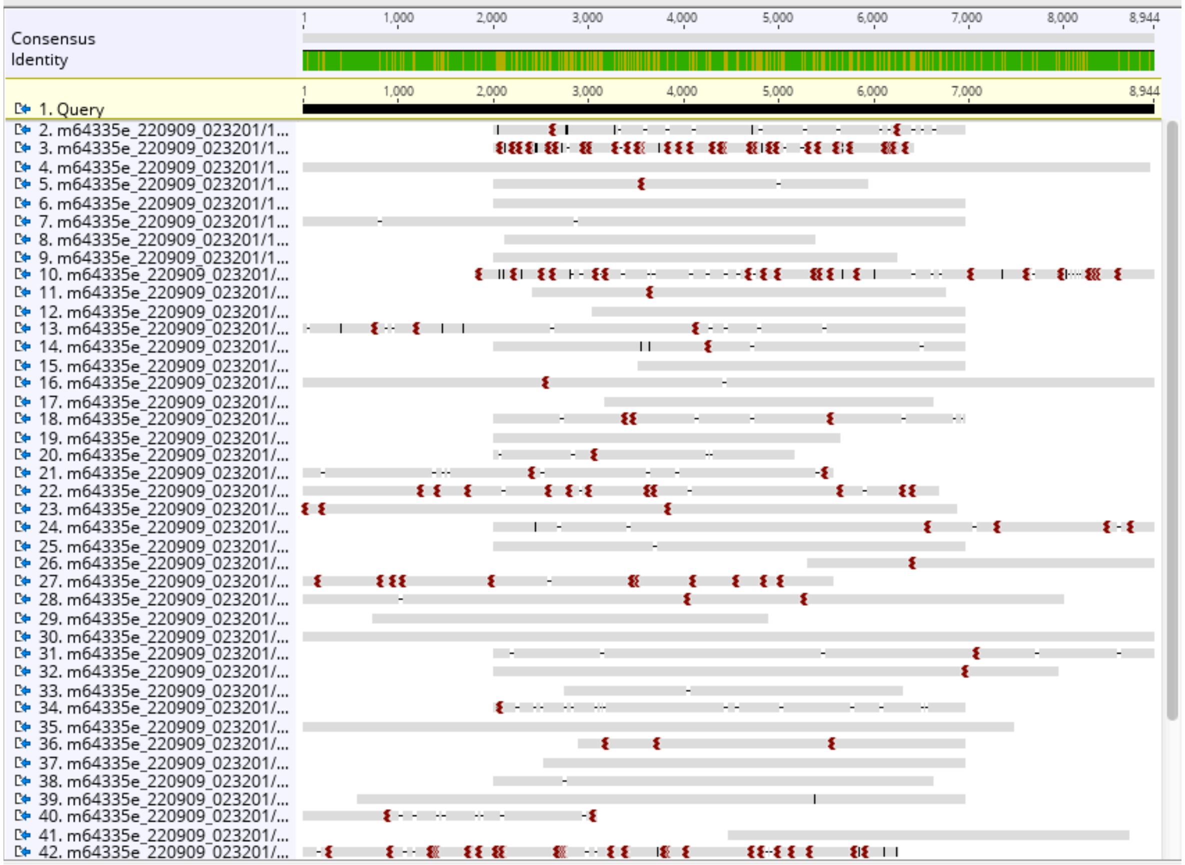

Figure S17. The PacBio HiFi reads of the type 1 individual Sample 32 were mapped to the fragment N-O-N (2000 bp sequences were taken from both ends of node N, with node O in the middle) by BLAST in Geneious 9. Reads spanning the entire fragment N-O-N can be seen, indicating the existence of a closed subgenomic ring consisting only of nodes N and O in type 1 individual Sample 32. Furthermore, reads from chromosome M1 were observed to map only to node O in the middle, and also reads were seen to map to node O and one end of node N (the other end entered chromosome M1, not shown here), representing a combination of the two subgenomic structures.

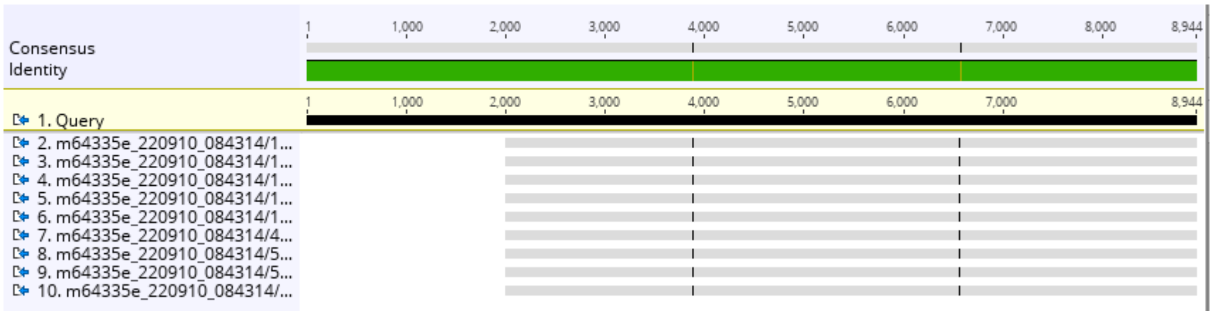

Figure S18. The PacBio HiFi reads of the type 2 individual Sample 4 were mapped to the fragment N-O-N (2000 bp sequences were taken from both ends of node N, with node O in the middle) by BLAST in Geneious 9. Node O was only connected to one end of node N but not to the other. The closed ring consisting only of node N and O does not exist in type 2 individual Sample 4.

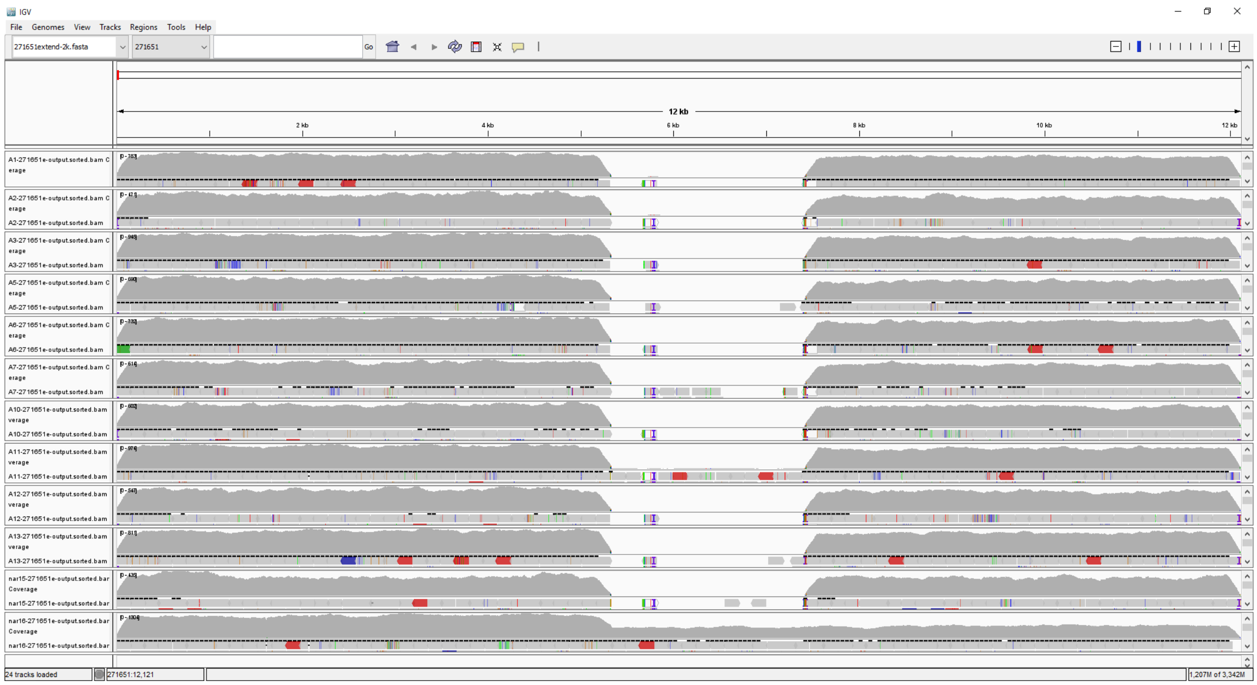

Figure S19. Bowtie 2 alignment of WGS Illumina reads from 12 marama samples to the 12,113 bp fragment from the type 2 mitochondrial genome chromosome M2, visualized in IGV. The reference sequence starts at C1 and ends at B (Figure 1), with the 2,108 bp type 2 exclusive sequence in between, from 5,325 bp to 7,432 bp. The 10 A plants and nar15, all originally from Aminuis, do not contain this 2,108 bp fragment, indicating that they have a type 1 mitochondrial genome. Furthermore, in these plants, the coverage of C1 is close to that of B, as expected for the type 1 structure. nar16 is a descendent of plants grown on Pretoria Farm and has a type 2 mitochondrial genome. Not only does it contain this 2,108 bp fragment, but it also has a depth-doubled C1 because that’s a repeat sequence that both M2 and M3 have, as shown in Figure 1.

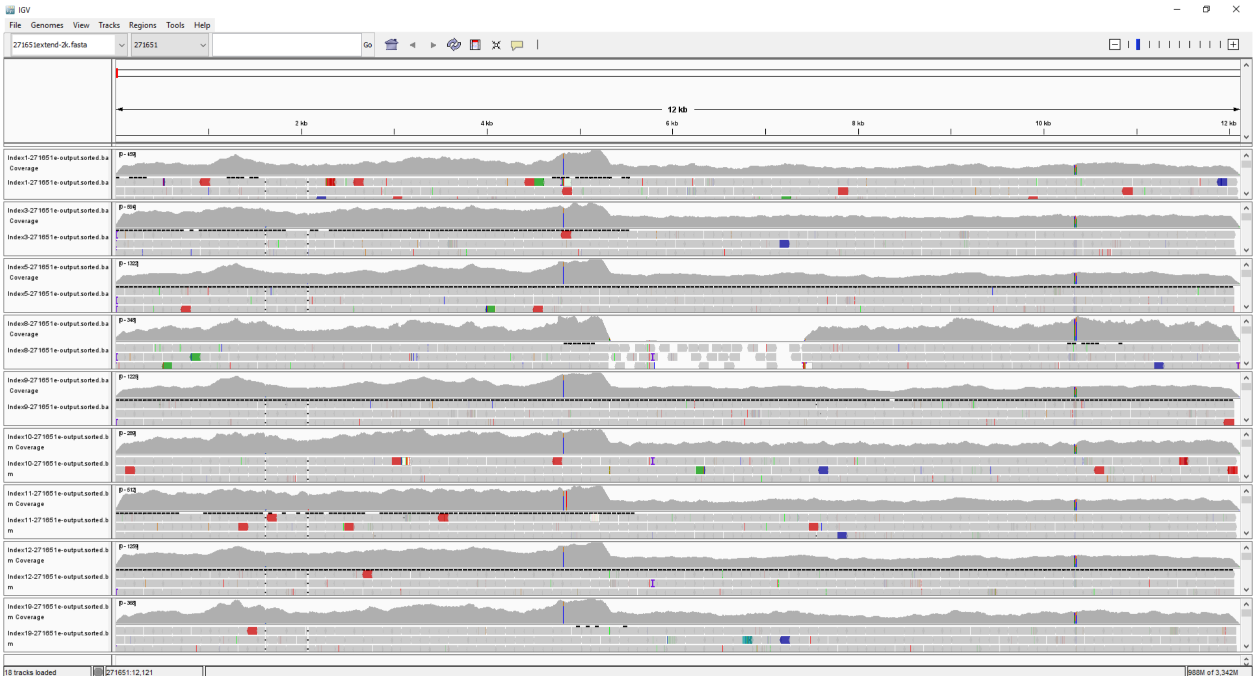

Figure S20. Bowtie 2 alignment of WGS Illumina reads from 9 Index plants to the 12,113 bp fragment from the type 2 mitochondrial genome chromosome M2, visualized in IGV. The reference sequence starts at C1 and ends at B (Figure 1), with the 2,108 bp type 2 specific sequence in between, from 5,325 bp to 7,432 bp. Among these plants, Index8, as the only one not originating from the Pretoria Farm, does not contain this 2,108 bp fragment, indicating that it has a type 1 mitochondrial genome. All remaining Index plants were either collected from the Pretoria Farm or were grown from seeds collected there. They all have type 2 mitochondrial genomes and they all contain this 2,108 bp fragment. Besides, the depth of C1 is doubled in all these plants except Index8 because C1 is a repeat sequence that both M2 and M3 have in the type 2 mitogenome, as shown in Figure 1.

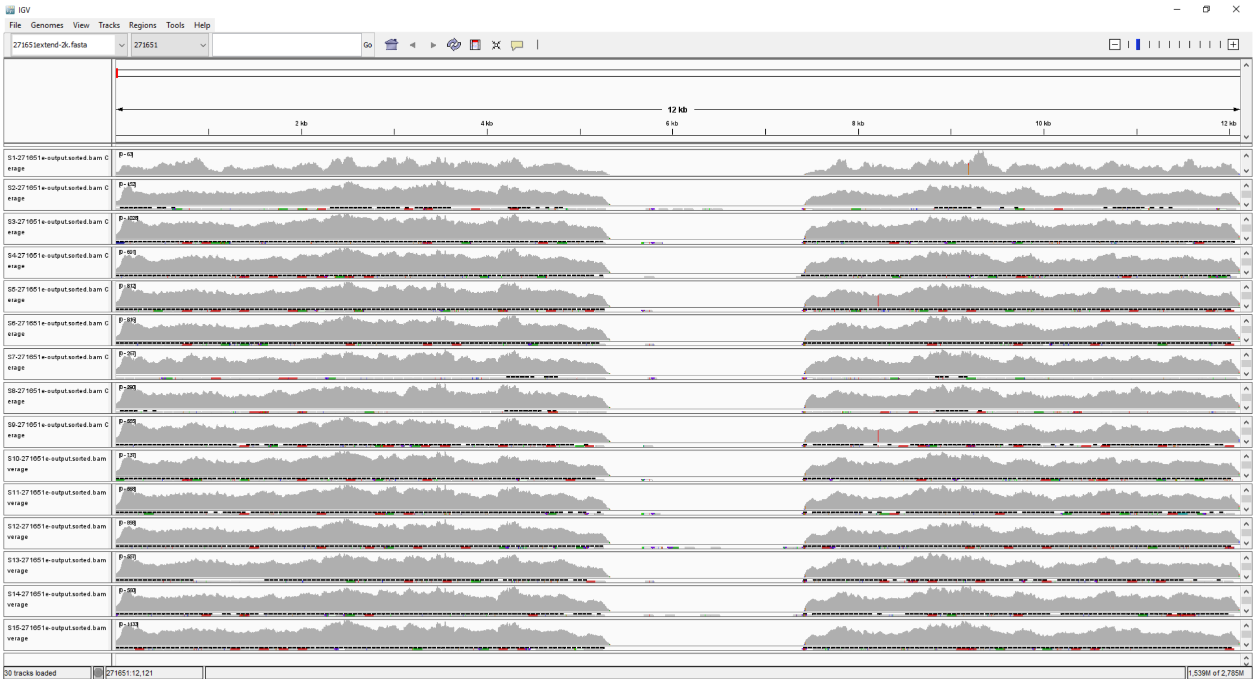

Figure S21. Bowtie 2 alignment of WGS Illumina reads from 15 S plants (S1-S15) to the 12,113 bp fragment from the type 2 mitochondrial genome chromosome M2, visualized in IGV. The reference sequence starts at C1 and ends at B (Figure 1), with the 2,108 bp type 2 specific sequence in between, from 5,325 bp to 7,432 bp. S plants are wild plants collected from 8 different geographical locations in Namibia. None of them contain this 2,108 bp fragment, indicating that they all have the type 1 mitochondrial genome.

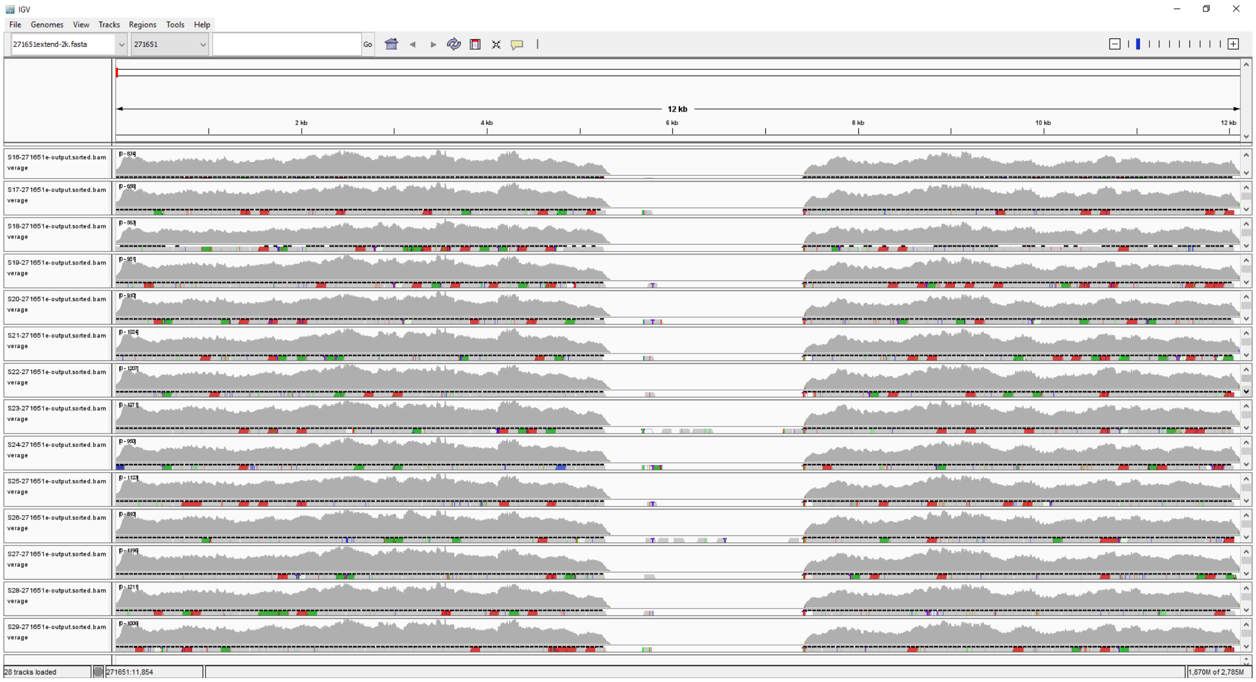

Figure S22. Bowtie 2 alignment of WGS Illumina reads from 14 S plants (S16-S29) to the 12,113 bp fragment from the type 2 mitochondrial genome chromosome M2, visualized in IGV. The reference sequence starts at C1 and ends at B (Figure 1), with the 2,108 bp type 2 specific sequence in between, from 5,325 bp to 7,432 bp. S plants are wild plants collected from 8 different geographical locations in Namibia. None of them contain this 2,108 bp fragment, indicating that they all have the type 1 mitochondrial genome.

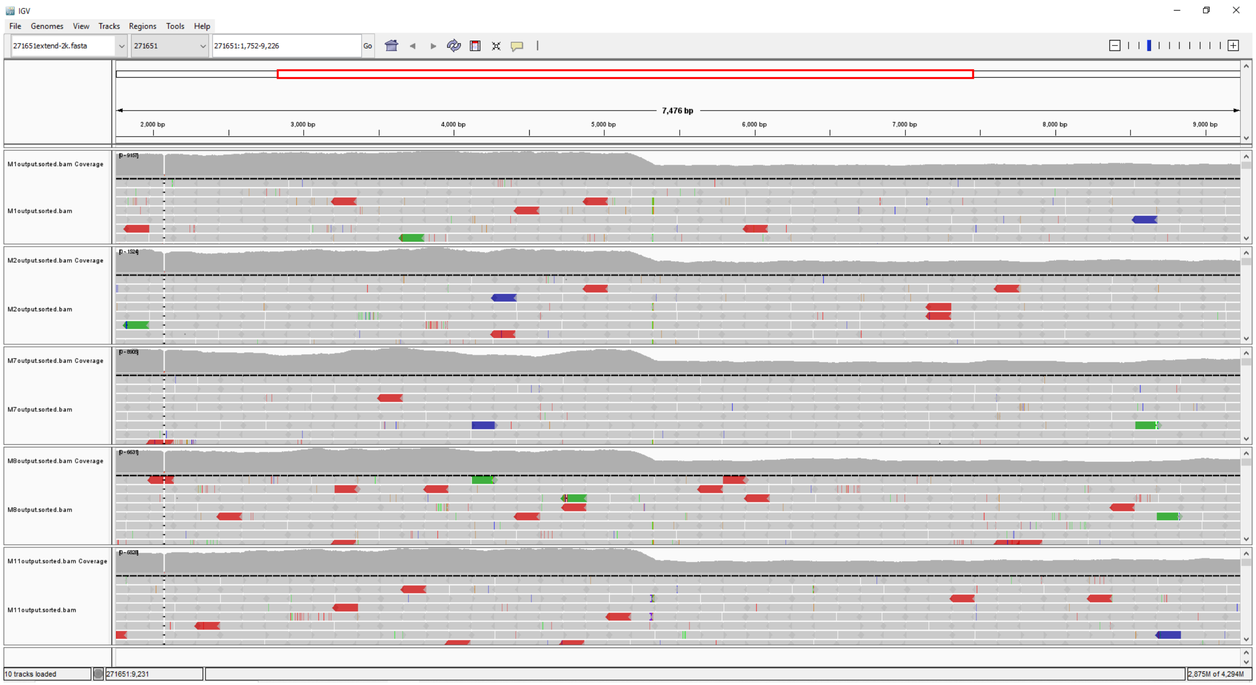

Figure S23. Bowtie 2 alignment of WGS Illumina reads from 5 M plants (M1, M2, M7, M8, and M11) to the 12,113 bp fragment from the type 2 mitochondrial genome chromosome M2, visualized in IGV. The reference sequence starts at C1 and ends at B (Figure 1), with the 2,108 bp type 2 specific sequence in between, from 5,325 bp to 7,432 bp. M plants (except M40) were grown from seeds collected from Namibia Farm. They all contain this 2,108 bp fragment, indicating that they all have the type 2 mitochondrial genome. Furthermore, the coverage of the C1 region is doubled in these plants because it is a repeat sequence that both chromosomes M2 and M3 have in the type 2 mitogenome, as shown in Figure 1.

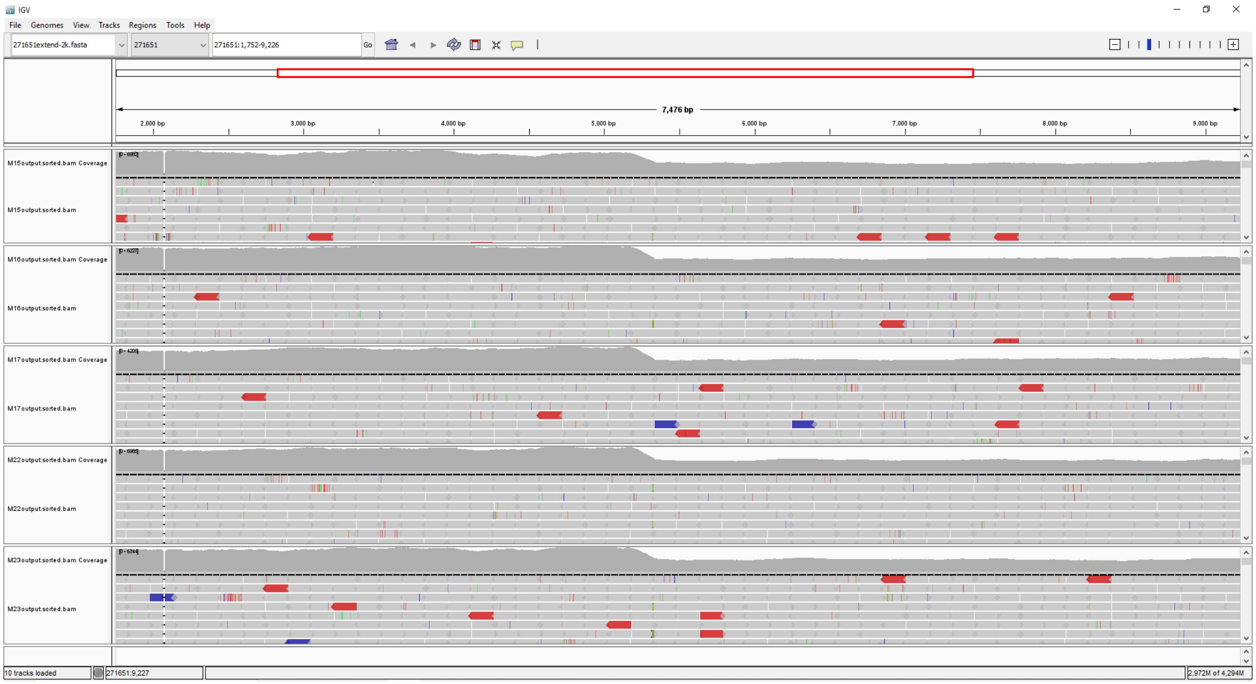

Figure S24. Bowtie 2 alignment of WGS Illumina reads from 5 M plants (M15, M16, M17, M22, and M23) to the 12,113 bp fragment from the type 2 mitochondrial genome chromosome M2, visualized in IGV. The reference sequence starts at C1 and ends at B (Figure 1), with the 2,108 bp type 2 specific sequence in between, from 5,325 bp to 7,432 bp. M plants (except M40) were grown from seeds collected from Namibia Farm. They all contain this 2,108 bp fragment, indicating that they all have type 2 mitochondrial genomes. Furthermore, the coverage of the C1 region is doubled in these 5 plants because it is a repeat sequence that both chromosomes M2 and M3 have in the type 2 mitogenome, as shown in Figure 1.

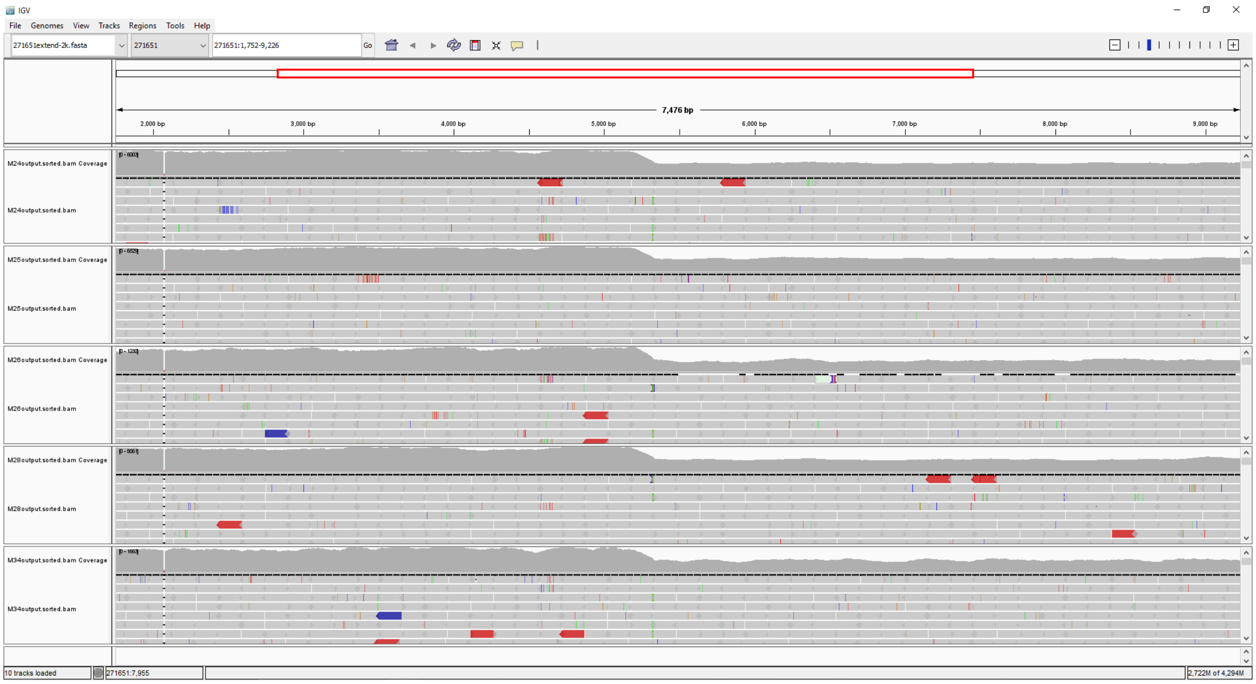

Figure S25. Bowtie 2 alignment of WGS Illumina reads from 5 M plants (M24, M25, M26, M28, and M34) to the 12,113 bp fragment from the type 2 mitochondrial genome chromosome M2, visualized in IGV. The reference sequence starts at C1 and ends at B (Figure 1), with the 2,108 bp type 2 specific sequence in between, from 5,325 bp to 7,432 bp. M plants (except M40) were grown from seeds collected from Namibia Farm. They all contain this 2,108 bp fragment, indicating that they all have type 2 mitochondrial genomes. Furthermore, the coverage of the C1 region is doubled in these plants because it is a repeat sequence that both chromosomes M2 and M3 have in the type 2 mitogenome, as shown in Figure 1.

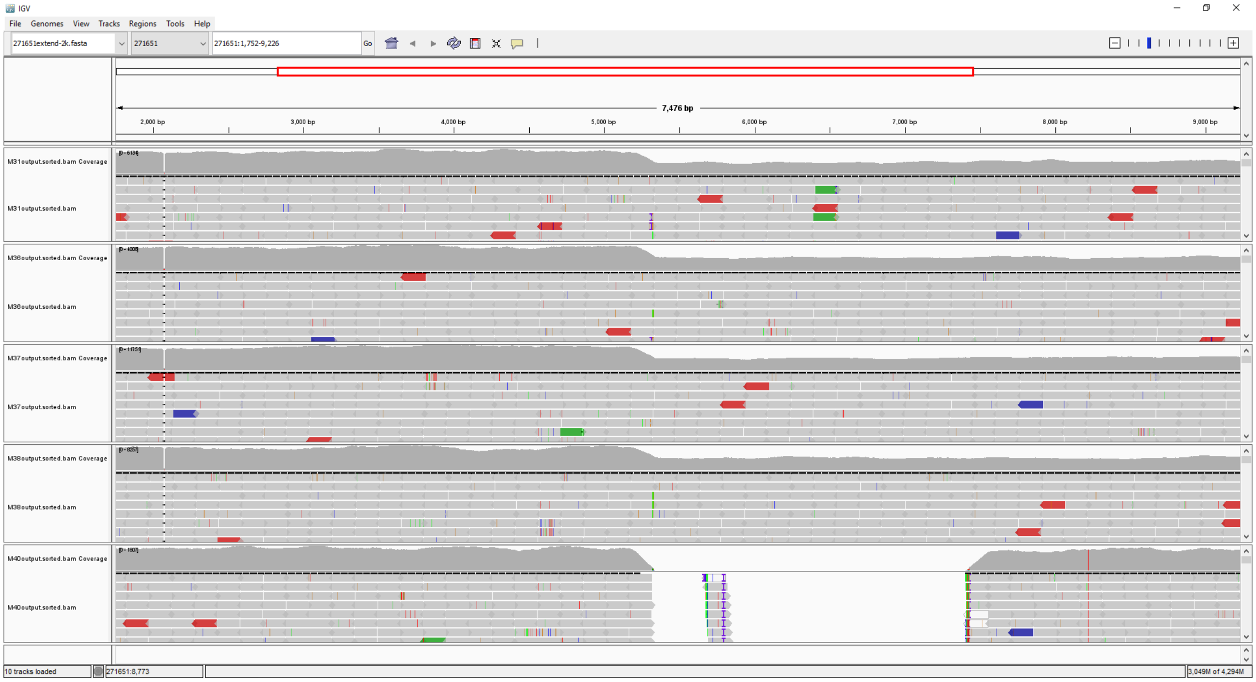

Figure S26. Bowtie 2 alignment of WGS Illumina reads from 5 M plants (M31, M36, M37, M38, and M40) to the 12,113 bp fragment from the type 2 mitochondrial genome chromosome M2, visualized in IGV. The reference sequence starts at C1 and ends at B (Figure 1), with the 2,108 bp type 2 specific sequence in between, from 5,325 bp to 7,432 bp. All M plants (except M40) were grown from seeds collected from Namibia Farm. The origin of sample M40 is unknown. All plants except M40 contain this 2,108 bp fragment, indicating that they have type 2 mitochondrial genomes. Furthermore, the coverage of the C1 region is doubled in these 4 plants because it is a repeat sequence that both chromosomes M2 and M3 have in the type 2 mitogenome, as shown in Figure 1. M40 doesn’t have this 2,108 bp fragment, and its C1 and B have close sequencing coverage, suggesting that M40 has a type 1 mitogenome.

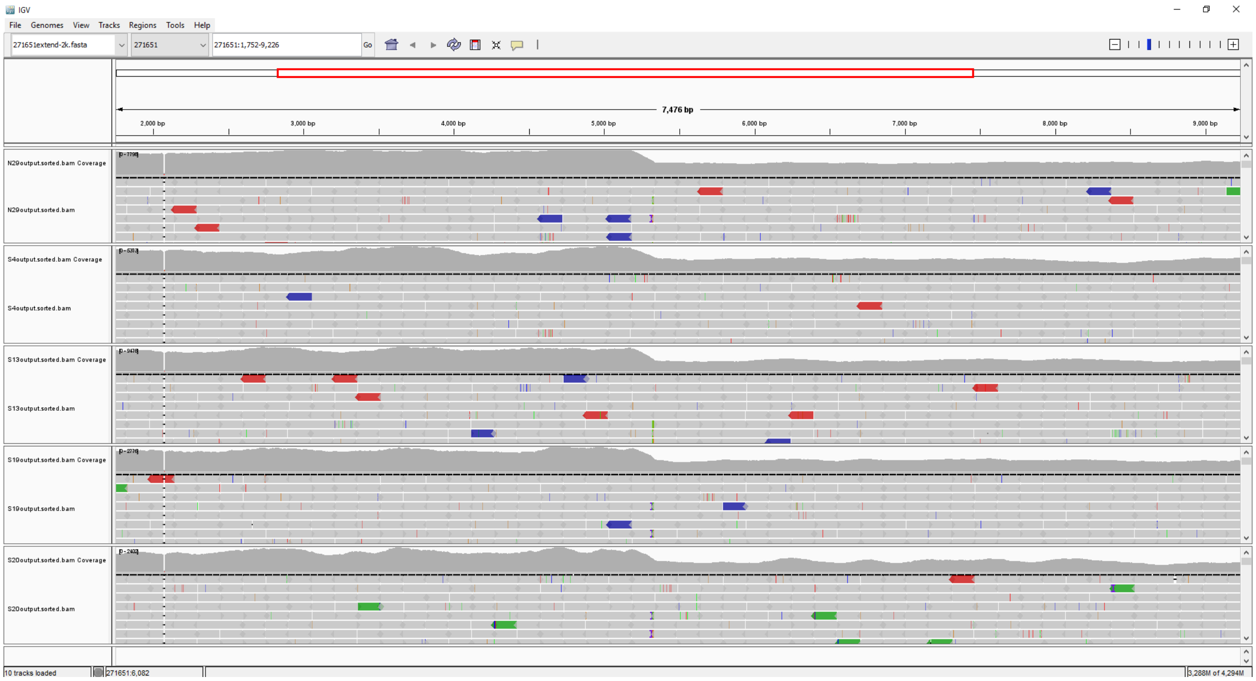

Figure S27. Bowtie 2 alignment of WGS Illumina reads from 5 M individuals (N29, S_4, S_13, S_19, and S_20) to the 12,113 bp fragment from the type 2 mitochondrial genome chromosome M2, visualized in IGV. The reference sequence starts at C1 and ends at B (Figure 1), with the 2,108 bp type 2 specific sequence in between, from 5,325 bp to 7,432 bp. M plants (except M40) were grown from seeds collected from Namibia Farm. They all contain this 2,108 bp fragment, indicating that they all have type 2 mitochondrial genomes. Furthermore, the coverage of the C1 region is doubled in these plants because it is a repeat sequence that both chromosomes M2 and M3 have in the type 2 mitogenome, as shown in Figure 1.

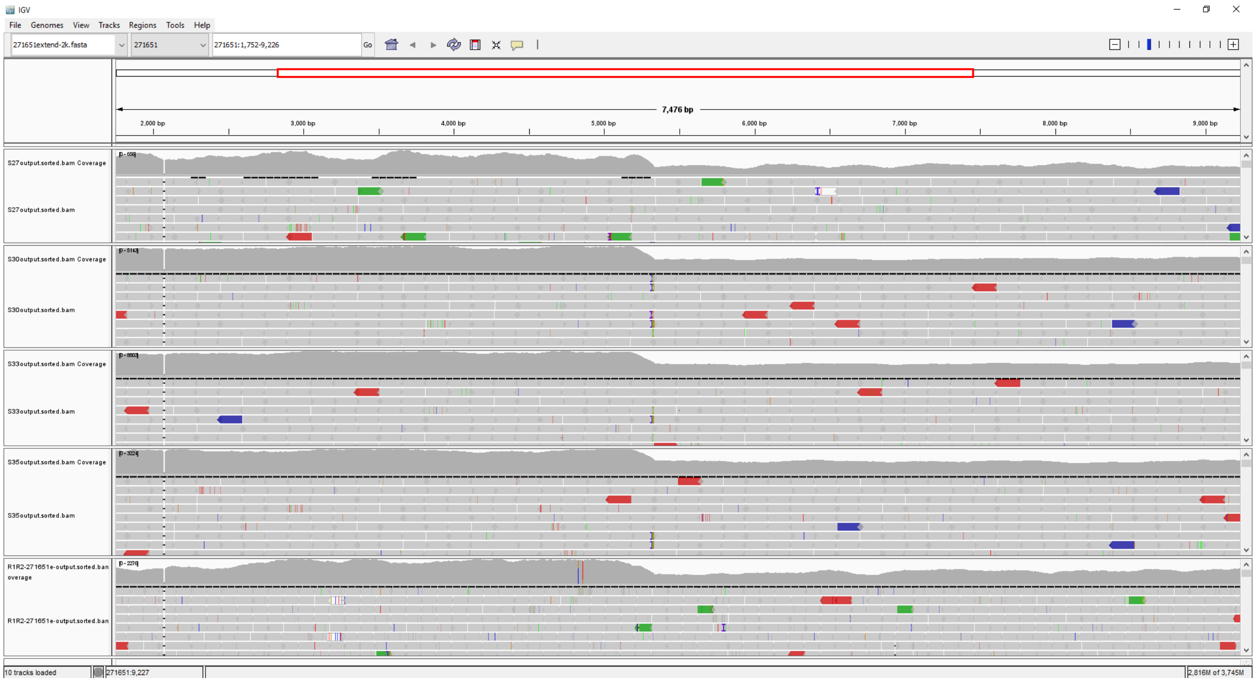

Figure S28. Bowtie 2 alignment of WGS Illumina reads from 4 M plants (S_27, S_30, S_33, and S_35) and R1R2 to the 12,113 bp fragment from the type 2 mitochondrial genome chromosome M2, visualized in IGV. The reference sequence starts at C1 and ends at B (Figure 1), with the 2,108 bp type 2 specific sequence in between, from 5,325 bp to 7,432 bp. M plants (except M40) were grown from seeds collected from Namibia Farm. The origin of sample R1R2 is unknown. However, they all contain this 2,108 bp fragment, indicating that they all have type 2 mitochondrial genomes. Furthermore, the coverage of the C1 region is doubled in all these 5 individuals, which consistent with the type 2 mitogenome structure, as C1 is a repeat sequence contained in both chromosomes M2 and M3 in the type 2 mitogenome, as shown in Figure 1.

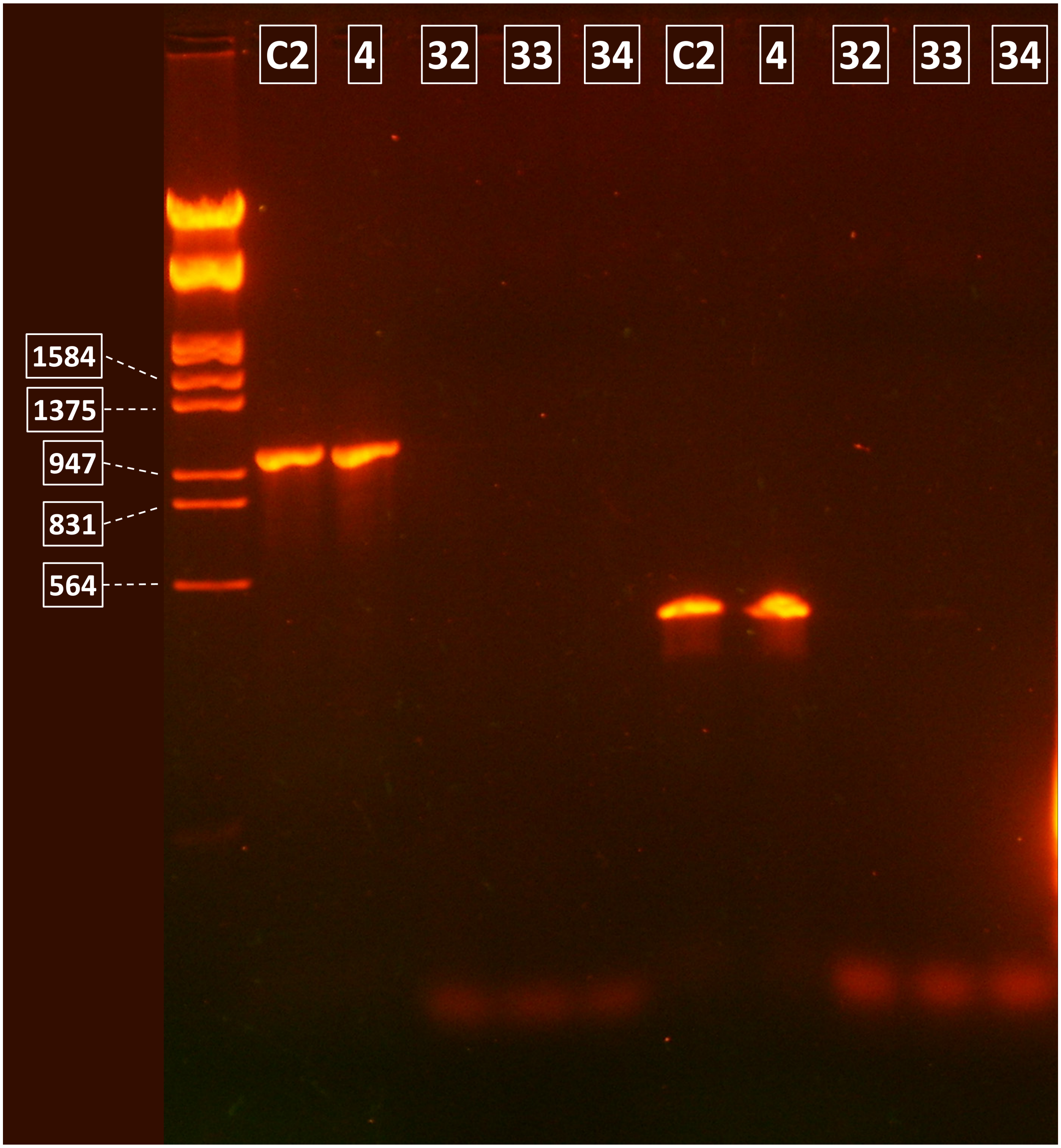

Figure S29. Gel image of DNA PCR amplification of five randomly selected samples C2, 4, 32, 33, and 34 using two pairs of primers designed to span both ends of the 2k type 2 mitogenome unique fragment. Lane1, Lambda DNA/EcoRI + HindIII Marker; Lanes 2 and 7, sample C2; Lanes 3 and 8, sample 4; Lanes 4 and 9, sample 32; Lanes 5 and 10, sample 33; Lanes 6 and 11, sample 34; The products in lanes 2 to 6 were amplified with the 2k left forward and reverse primers, in lane 7 to 11 were amplified with the 2k right forward and reverse primers. The PCR products were electrophoresed on a 1.5% agarose gel at 110V for 2 hours.

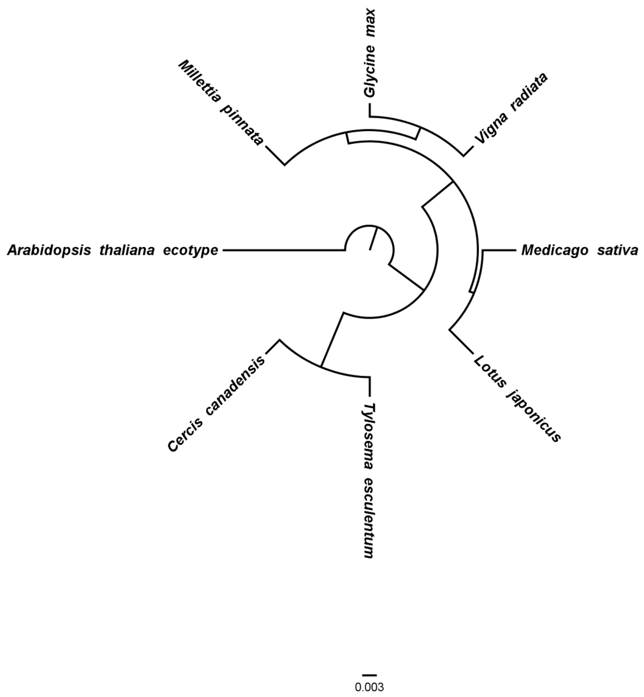

Figure 30. Bayesian inference tree on artificial chromosomes concatenated by 24 conserved mitochondrial genes, *atp1*, *atp4*, *atp6*, *atp8*, *atp9*, *nad3*, *nad4*, *nad4L*, *nad6*, *nad7*, *nad9*, *mttB*, *matR*, *cox1*, *cox3*, *cob*, *ccmFn*, *ccmFc*, *ccmC*, *ccmB*, *rps3*, *rps4*, *rps12*, and *rpl16* from the mitochondrial genomes of *Arabidopsis thaliana* (NC_037304.1), *Cercis canadensis* (MN017226.1), *Lotus japonicus* (NC_016743.2), *Medicago sativa* (ON782580.1), *Millettia pinnata* (NC_016742.1), *Glycine max* (NC_020455.1), and *Vigna radiata* (NC_015121.1) in NCBI drawn by BEAST and FigTree.

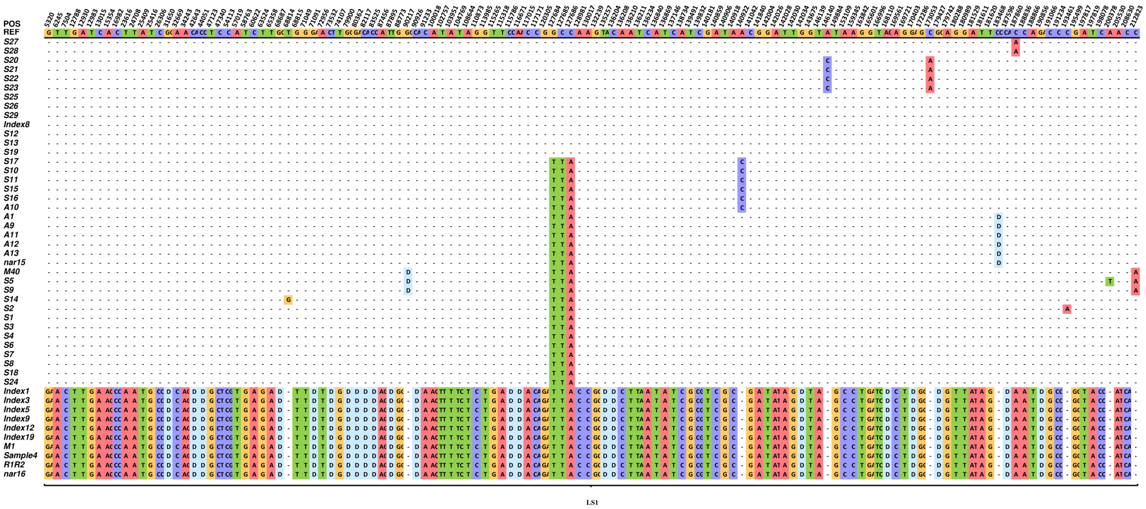

Figure S31. Distribution of variations discovered when aligning the WGS reads of 38 type 1 individuals and 10 type 2 plants to the first 212,823 bp of the marama reference mitogenome

chromosome LS1, OK638188. The first row shows the bases in the reference sequence, from the second row onwards only the bases that differ from the reference are shown, and the bases that are the same as the reference are replaced by dashes. All insertions are represented by the first two bases and deletions by the letter “D”.

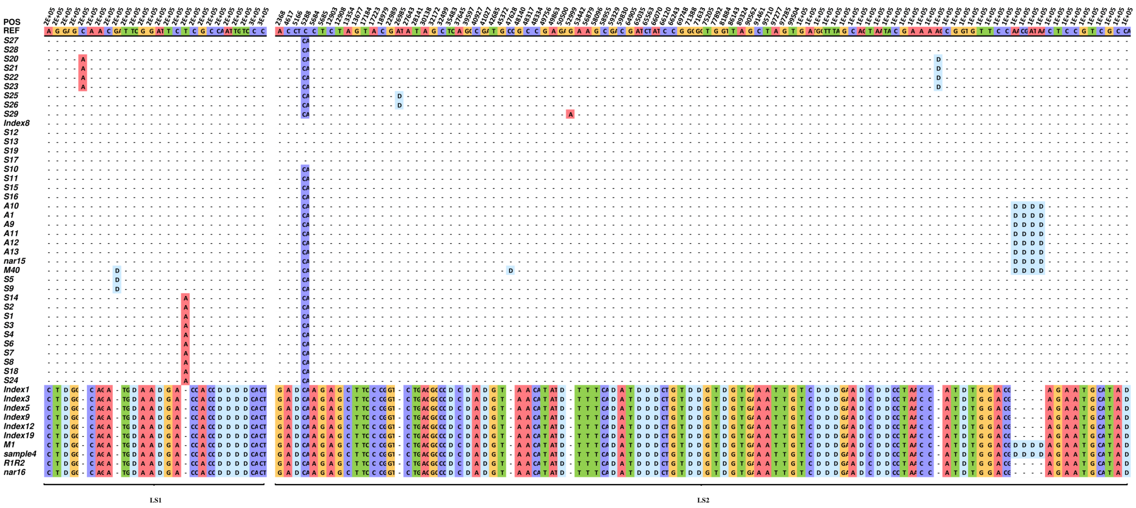

Figure S32. Distribution of variations discovered when aligning the WGS reads of 38 type 1 individuals and 10 type 2 plants to the marama reference mitogenome chromosomes LS1 (OK638188, 212,823 bp to 253,259 bp) and LS2 (OK638189). The first row shows the bases in the reference sequence, from the second row onwards only the bases that differ from the reference are shown, and the bases that are the same as the reference are indicated by dashes. All insertions are represented by the first two bases and deletions by the letter “D”.

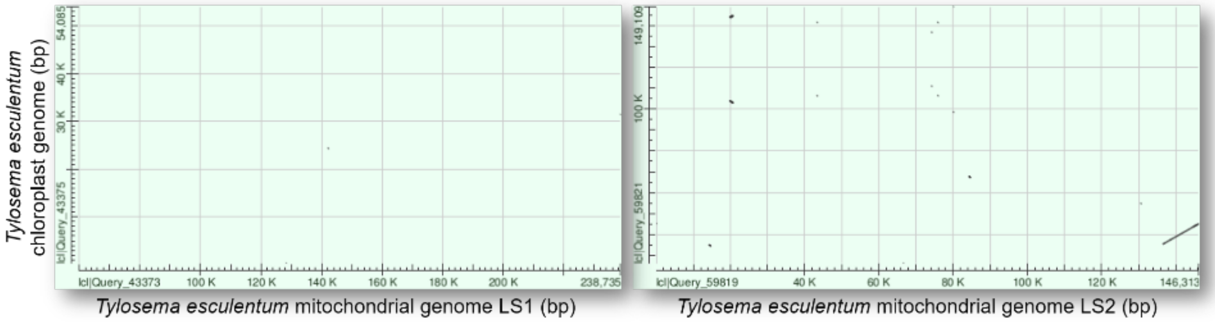

Figure S33. A dot plot view showing the alignment of marama mitochondrial chromosomes to the chloroplast genome. The two reference mitochondrial chromosomes of marama (OK638188 and OK638189) were blasted against the reference chloroplast genome of marama (KX792933.1), respectively in NCBI.

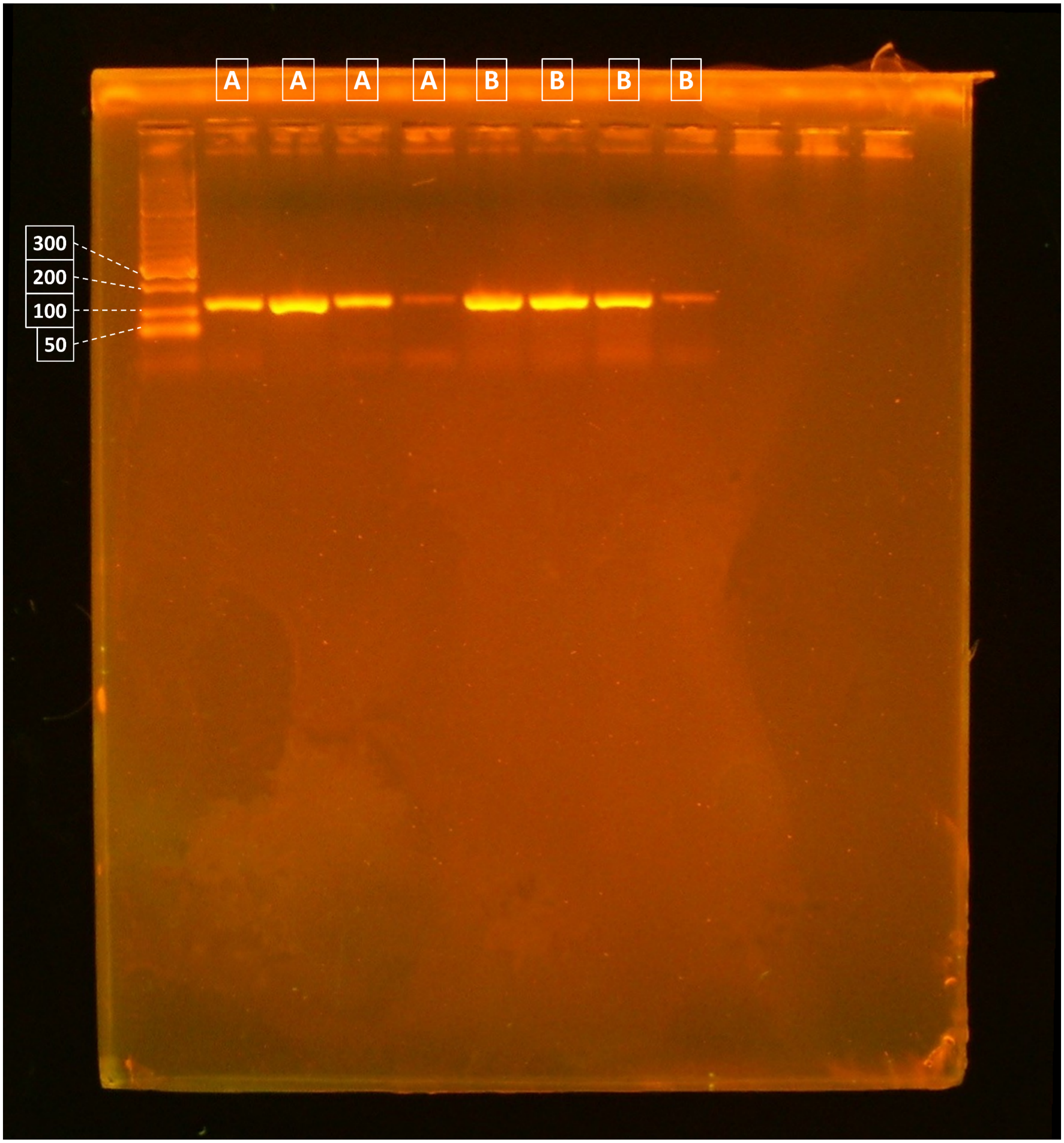

Figure S34. Amplification across the two ends of the 9,798 bp homologous fragment of the mitochondrial and chloroplast genomes in two random samples A and B. Lane1, HyperLadder II; Lanes 2-5, DNA from sample A; Lanes 6-9, DNA from sample B. The products in lanes 2 and 6 were amplified with primers MitoLL and MitoLR, in lane 3 and 7 were amplified with primers ChlLL and MitoLR, in lanes 4 and 8 were amplified with primers MitoRL and MitoRR, and in lanes 5 and 9 were amplified with primers MitoRL and ChlRR. The gel was run on a 1.5% agarose gel for 40 min at 100V. The annealing temperature of 55 °C was a bit high for the primer

ChlRR, resulting in low yields and faint bands in lanes 5 and 9. Decreasing the annealing temperature by 1 °C and repeating the PCR resulted in normal amplification.
